# Energy Harvester Sensing

**DOI:** 10.3390/s20071849

**Published:** 2020-03-26

**Authors:** Fabio Viola

**Affiliations:** Department of Engineering, University of Palermo, 90128 Palermo, Italy; fabio.viola@unipa.it

## 1. Introduction

Our existence is immersed in a solution of energy, which is often left to degrade because we perceive this energy as expendable. The energy carrier is always widespread in our environments, we use the energy of light to control the behavior of small system. Sometimes, we are used to an abundance of energy, which, in its absence, we believe that certain operations cannot be carried out.

A thought must be made. If an object has an interest for its energy status, its own energy can be used to determine its parameters. 

In Greek mythology, Prometheus, is remembered for having deceived the gods of Olympus and bringing fire to mankind after he was abducted by Zeus, who wanted to punish human beings for an affront, leaving them devoid of a visual sense. 

Now researchers, as the modern Prometheus, can bring energy into every device for sensing using energy harvesting. In the case of systems where photovoltaic and wired sources are precluded, the use of energy harvesters, devices of very high conception, capable of capturing small sources of energy, transducing, and conditioning them into an electrical signal to be used by micro-sensors, is the right response.

Moreover, the research is moving toward the collection of multiple forms of information, which is not one set of values over the same and different times, but a set of values in knowledge, in the broadest sense of the term—the real-world objects. To represent an object, we can no longer rely on its secondary aspects but must have information on its behavior under stressful conditions. There are many sensors to be used, and the technologies that feed them must be less invasive. We therefore need to move from the vision of Plato’s shadows projected into a cave, to the true knowledge of things.

The term energy harvesting sensing identifies the innovation that allows us to increase the presence of sensors in all environments without having the use of energy supports such as replaceable batteries or directly hardwire power supply. In order to obtain similar results, a synergy between transducers, conditioning circuits, and sensors is necessary.

New energy harvesting devices based on electromagnetic or mechanical principles are continually being invented, exploiting electronic components to make power available for sensors.

## 2. Summary of Special Issue Papers

The main theme of the research on energy harvester sensors is represented by the low amount of energy. We can therefore distinguish some articles in which the interest of research is on the energy source and the transducer [1,2,3,4], from articles in which the interest is on how to maximize this extracted energy [5,6] or on how it is possible to use these small quantities of energy [7,8,9].

In [1], Doria et al. remind us that on a sunny day, the best energy source is solar power, but in the absence of the sun, we should not be discouraged but use systems that can collect kinetic energy from raindrops and convert it efficiently. Doria’s study differs from others that deal with the same topic, for the rigor in describing the behavior of the cantilever in the presence of water substrates. This study helps to illustrate the limitations of these transducers and to direct subsequent researchers to identify the best choices for connecting the hardware that will power the sensors.

In [2,3], Zhang et al. present research on nonlinear vibration energy harvesting techniques by showing the scientific community new mechanical devices: an arc-shaped piezoelectric bistable vibration energy harvester (ABEH) and a tri-stable piezoelectric vibration energy harvester based on a composite shape beam (TPEH-C). The presented devices are first analyzed in detail by means of a well-developed analytical model and are then tested experimentally. The main contribution, given to the scientific community, deals with the innovation in employing magnets in the piezoelectric transducers.

In [4], Su et al. perform a study on a piezoelectric cantilever with a tip mass subjected to a horizontal rotational motion. Rotational motion is a consequence of centrifugal force, which causes an axial load on the beam and alters the resonant frequency of the system. This point of view has never been considered by researchers.

In [5], Gong et al. put their attention on the second block of the energy harvesting sensing: the electrical architecture to be used to feed sensors. They propose a new circuit to replace the traditional diode circuit implementation in wireless power supply systems, applied wearable and implantable biomedical electronic devices, and sensing monitors. The article explains how to use very low consumption devices, making different comparisons with other circuit types.

In [6], Batista et al. move away from vibrational energy and embrace the extraction of energy from indoor photovoltaic sources. In the article, they explain the use an organic photovoltaic cell and a DC-DC converter, designed with a 130 nm complementary metal-oxide-semiconductor (CMOS) component, which reduce consumption and maximize power extraction. 

Gljušćić et al. in [7] change the game rules; they start from the power requirements of wearable sensors for medical applications and address the research of piezoelectric kinetic energy harvesting devices that can be used to power them by considering the narrow area of optimal operation around the eigenfrequencies of a specific device, the optimized harvester geometry, and the electrical circuitry required for efficient energy management.

Citroni at al. in [8] propose research of which the goals are to explore and develop new strategies to extend mission parameters of a new class of unmanned vehicles, named micro air vehicles (MAVs). They presented a complete analytical model, identifying different factors, which determine the MAV power consumption. The design of a nanoarray energy harvester, based on plasmonics nano-antenna technology, is proposed.

In [9], Shi et al. investigate secure communications of energy harvesting untrusted relay networks. Again, it is an application of energy harvesting sensing. Destination assists jamming signal to prevent the untrusted relay from eavesdropping and to improve the forwarding ability of the energy-constrained relay. 

In summary, energy harvesting sensing research is very active and interesting. The topics vary from the description of vibrational and optical transducers, with circuit architecture for the management of electricity, to applications in the sensing field.

In this sense, this special collection issue represents a dedicated survey of advances in all topics linked to energy harvesting sensing from a simple source of energy, e.g., a raindrop, to various applications in research and industry.
